# Thermal ecosystem engineering by songbirds promotes a symbiotic relationship with ants

**DOI:** 10.1038/s41598-020-77360-z

**Published:** 2020-11-23

**Authors:** Marta Maziarz, Richard K. Broughton, Luca Pietro Casacci, Anna Dubiec, István Maák, Magdalena Witek

**Affiliations:** 1 Museum and Institute of Zoology, Polish Academy of Sciences, Wilcza 64, 00-679 Warsaw, Poland; 2 UK Centre for Ecology & Hydrology, Maclean Building, Benson Lane, Crowmarsh Gifford, Wallingford, OX10 8BB UK; 3 Department of Life Sciences and Systems Biology, University of Turin, Via Accademia Albertina 13, 10123 Turin, Italy; 4 Department of Ecology, University of Szeged, Közép fasor 52, Szeged, 6726 Hungary

**Keywords:** Behavioural ecology, Community ecology, Ecological networks

## Abstract

Nesting birds can act as thermal ecosystem engineers by providing warm habitats that may attract arthropods to colonise the nest structure. This cohabitation of birds and nest-dwelling invertebrates may foster symbiotic relationships between them, but evidence is lacking. We investigated whether ants are attracted to bird nests by the heat generated by the hosts, and/or the nests’ structural insulation properties, to raise their broods (larvae and/or pupae) in advantageous thermal conditions. We found that the endothermic activity of birds within their nests created ‘heat islands’, with thermal conditions potentially promoting the survival and development of ant larvae in cool environments. We experimentally confirmed that the presence of heat within bird nests, and not the structure itself, attracted the ants to colonise the nests. As ants might benefit from exploiting warm bird nests, this may be a previously overlooked commensal, mutualistic or parasitic relationship which may be ecologically significant and globally widespread among various nesting birds and reproducing ants. Similar interspecific interactions may exist with other arthropods that reproduce in avian and mammalian nests. Further research is needed to reveal the nature of these relationships between such taxa, and to understand the role of warm-blooded animals as thermal ecosystem engineers.

## Introduction

Many animals build structures that can also be used by other organisms. These builders act as ‘ecosystem engineers’ by transforming the physical environment and creating, modifying and/or maintaining habitats for other species^1,2^. Well-known examples of ecosystem engineering include beavers (Castoridae) damming streams to create wetlands that are inhabited by a diverse community of plants and animals^3^, or burrowing mammals that change the physical and chemical properties of soil available for plants^4,5^. Nesting birds are another example of a diverse group of ecosystem engineers, which are widespread throughout the world. By excavating tree-cavities, burrowing and/or building their nests on or above the ground, birds create novel habitats exploited by a wide spectrum of plants and animals, including invertebrates, amphibians, lizards and mammals^6^.

Bird nests host a large diversity of colonising arthropods (e.g.^7–12^ and references therein). Although the structures built by birds can function as shelters or a food resource for nest-dwelling arthropods, they may also provide desirable warm microclimates. Bird nests are often composed of insulating materials that protect the parents and their eggs or nestlings from excessive heat loss^13,14^, and they are heated from within by brooding adults or fully feathered chicks^15–18^. Thus, active nests that are currently occupied by birds might form well-insulated ‘heat islands’ in cold environments, which may be important for the enhanced survival and reproduction of nest-dwelling arthropods.

The role of nesting birds as ‘thermal ecosystem engineers’ was suggested by Sinclair and Chown^19^ in relation to wandering albatrosses *Diomedea exulans* and Marion flightless moths *Pringleophaga marioni* in the sub-Antarctic. The authors found that the insects appeared to gain a selective advantage by using active bird nests, where the survival of the moth larvae could be improved. Similar relationships might occur among other bird and arthropod taxa that cohabit in bird nests, but studies are lacking.

Close cohabitation of birds and arthropods has the potential to enable symbiotic relationships between the two groups, but limited investigation has resulted in few documented examples. Most research into the fauna of bird nests has concentrated on the relationships with blood-sucking ectoparasites, such as blow flies (Diptera, Calliphoridae) or fleas (Siphonaptera), with little attention paid to other nonparasitic invertebrate groups. As such, more research is needed to examine the relationships between birds and other invertebrate groups. Investigations are also required to clarify whether thermal engineering by birds may support undetected symbiotic (commensal, mutualistic or parasitic) relationships with nest-dwelling arthropods, which could benefit from raising their broods within warm bird nests.

We address these questions by exploring a previously unknown relationship between ground-nesting wood warblers *Phylloscopus sibilatrix*^20^ and ants, primarily *Myrmica ruginodis* and *M. rubra,* raising their broods (larvae and/or pupae) in the birds’ nests. Few studies have reported ant broods in bird nests, presumably because such cases have been frequently overlooked, and the mechanism of ant colonisation of bird nests remains unclear^21,22^. Limited information on the prevalence of ant broods in bird nests comes from Europe and the Americas, where the high occurrence of ant broods in the nests of some common birds (over 20%) suggest interspecific attraction, with at least one side of the system gaining an advantage from the association^21–28^.

The mechanism underpinning nest colonisation by ants could involve exploitation of the advantageous thermal conditions provided by the structural properties of bird nests, and/or the body heat from the avian hosts^26^. Access to a protein-rich food resource in the form of other arthropods inhabiting bird nests may also attract ant workers to forage in such places^22,24,29^. Nevertheless, a suitably warm microclimate could be a prime driver for ant workers to translocate their larvae into the warm structures of bird nests, reflecting the preference of many ant species for occupying warm nest locations^30^.

We hypothesise that *Myrmica* ants are attracted to bird nests to exploit the heat generated by the hosts and/or the enhanced insulation properties of the nest structure, to raise their broods in more advantageous (warmer and stable) thermal conditions than in the ants’ own nests. To test this hypothesis, we examine how the endothermic activity of birds modifies the thermal conditions of their nests in relation to the stage of the nesting cycle and/or ambient temperatures, possibly affecting the amount of heat generated by the birds^16,17,31,32^. Next, we explore whether thermal ecosystem engineering by the wood warblers may have potential benefits for ants raising their broods within the birds’ nests. Finally, we compare the insulative properties of the bird and ant nests, and test experimentally the ants’ attraction to heated and unheated bird nests to confirm whether the presence of heat, or the insulation of the nest structure itself, is important for ant colonisation.

## Results

### Birds as ‘thermal ecosystem engineers’

The presence of incubated bird eggs or older nestlings in active nests of wood warblers resulted in elevated temperatures of the nest walls, where ant workers and their broods occur^21^. Differences between mean daily internal and ambient temperatures were higher in active bird nests containing incubated eggs than in the same nests when vacated (means ± SD respectively 0.8 °C ± 1.3 and − 1.5 °C ± 0.9; paired t-test, t = 11.6, *P* < 0.001, n = 44 nests). This disparity was even greater, however, between active nests containing older nestlings and the vacant nests (mean daily internal-ambient temperature differences: means ± SD respectively 4.9 °C ± 2.6 and − 1.4 °C ± 0.9; paired t-test, t = 13.2, *P* < 0.001, n = 29 nests).

**Figure 1:**
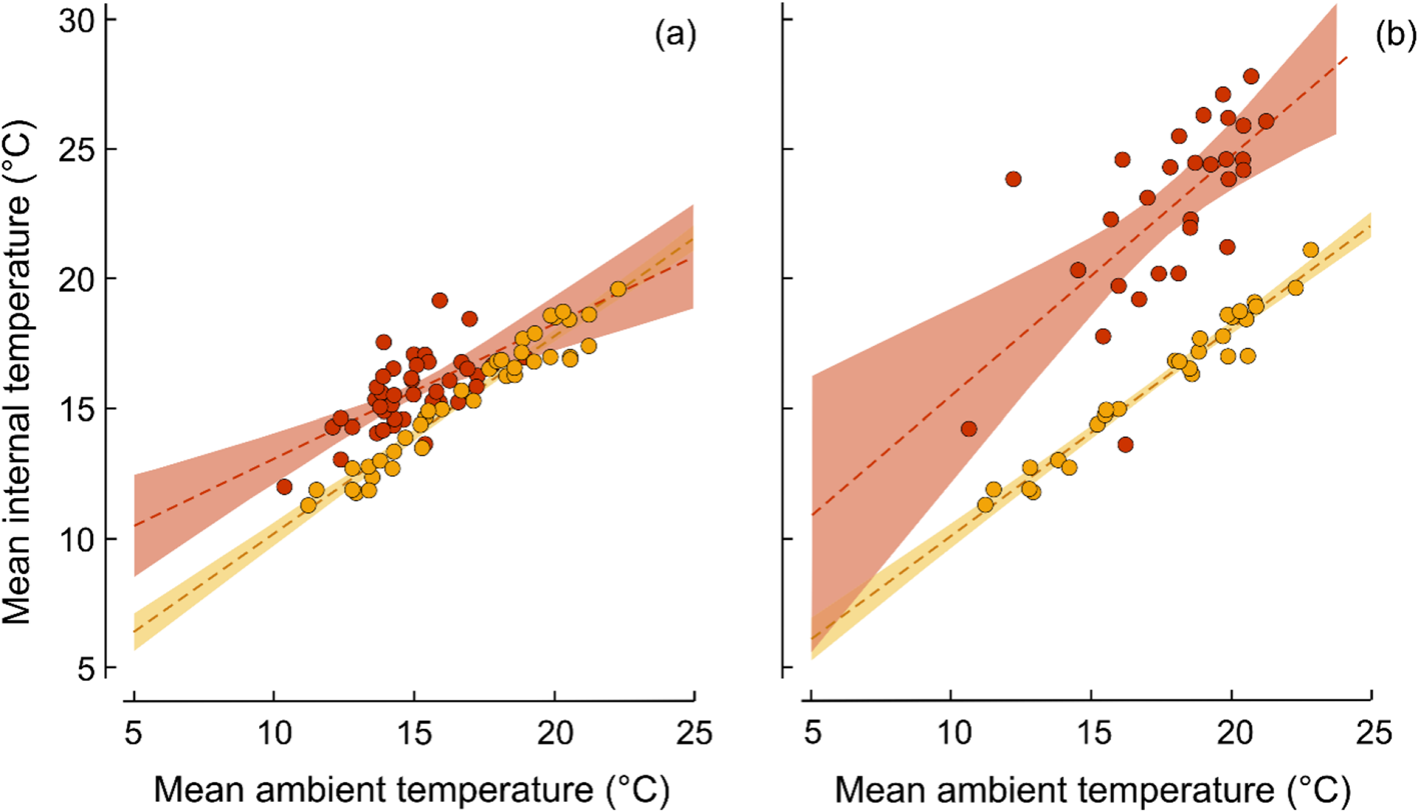
The relationship between mean daily ambient and internal temperatures in active (red circle) wood warbler *Phylloscopus sibilatrix* nests and in the same nests when vacant (yellow circle), recorded in 2017–2019 in the Białowieża National Park (Poland). Active nests contained incubated eggs (**a**; n = 44 nests), or nestlings of a median 7 days old (**b**; n = 29 nests). Dashed lines show trends and shaded areas are 95% confidence intervals.

The mean daily temperature of nest walls always increased with increasing ambient temperatures (Fig. 1a,b; Table 1). Nevertheless, the heating effect created by birds within their nests varied in relation to ambient conditions, but only during the egg incubation stage. Brooding birds warmed their nests more intensively on cold days than during warm weather, when the mean daily temperatures of the same active and vacant nests were more alike compared to low ambient temperatures (Fig. 1a; Table 1). By contrast, the temperatures of nests containing nestlings were consistently higher than for the same vacant nests, regardless of ambient conditions (Fig. 1b; Table 1).

**Table 1:** Results of bootstrapped (2000 replications) linear regressions showing the changes of mean daily internal temperature (response variable) with the covariates of respective ambient temperature and nest type: active (occupied) and vacant (inactive) nests of wood warblers *Phylloscopus sibilatrix* in the Białowieża National Park (Poland).

Variable	β	s.e.m	BCa CI	
			25%	75%
Active bird nests containing incubated eggs vs. vacant nests (n = 44 each)				
Intercept	**2.61**	**0.53**	**1.62**	**3.69**
Mean daily ambient temperature (°C)	**0.76**	**0.03**	**0.69**	**0.82**
Nest type (active)	**5.29**	**1.50**	**2.12**	**8.03**
Mean daily ambient temperature (°C) x nest type (active)	**− 0.24**	**0.10**	**− 0.42**	**− 0.01**
Active bird nests containing nestlings vs. vacant nests (n = 29 each)				
Intercept	1.35	1.62	− 1.31	5.11
Mean daily ambient temperature (°C)	**0.84**	**0.09**	**0.63**	**0.99**
Nest type (active)	**6.37**	**0.51**	**5.23**	**7.28**

Compared are active nests that contained incubated eggs, or nestlings of a median 7 days old, and the same nests when vacant (post-fledging or nest failure). Including the interaction term visibly improved the model fit only for the incubation stage (L = 7.1, df = 1, *P* = 0.008). The statistically significant results are highlighted with bold font. Bias-corrected and accelerated bootstrap confidence intervals (BCa CI) are shown.

### Thermal conditions in bird nests compared to ant nests

**Figure 2:**
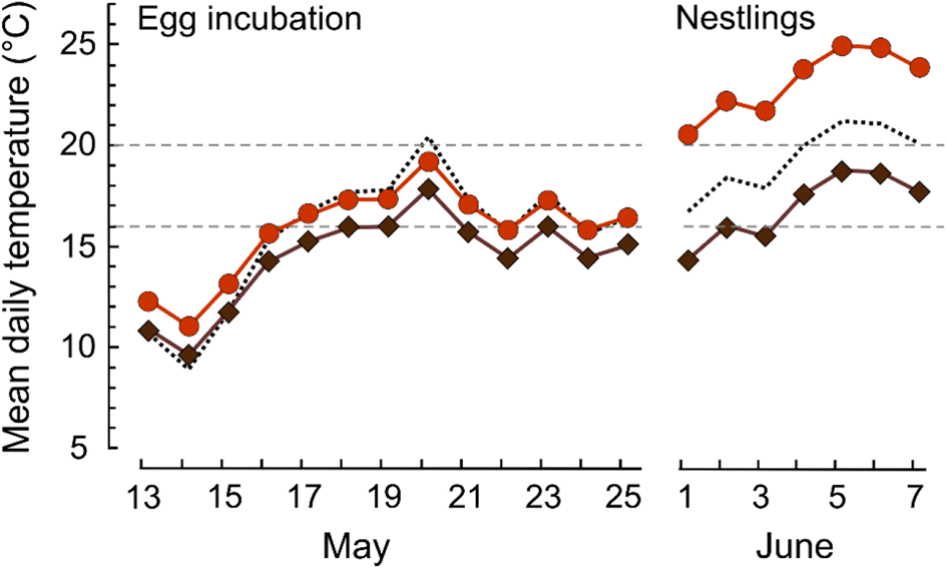
Modelled predictions of mean daily internal temperatures within wood warbler nests containing either incubated eggs or fully-feathered nestlings (red circle), and in *Myrmica* ant nests (brown diamond) at the same ambient temperatures (dotted line) recorded by the meteorological station in Białowieża village in 2019. Values shown are the ambient and predicted internal temperatures for the periods corresponding to the egg incubation (left) and the later nestling period (right) for the majority of wood warbler nests monitored in 2019. The two horizontal (dashed) lines mark the minimum temperature (16 °C) and lower threshold of the optimum temperature (20–25 °C) necessary for the development of *Myrmica* ant broods^33^.

Actively heated bird nests containing eggs or nestlings provided more advantageous thermal conditions for ant broods than the ants’ own nests. Based on mean daily ambient temperatures recorded during 2019 for the periods, when incubated eggs and older nestlings were present in most wood warbler nests, modelled predictions of temperatures were always much higher for within the walls of active bird nests than for ant nests (Fig. 2; Table 2).

**Table 2:** Results of bootstrapped (2000 replications) linear regression showing the changes of mean daily internal temperature (response variable) with main effects of the respective ambient temperature and nest type (wood warbler nests containing incubated eggs, or nestlings of a median 7 days old, vs. *Myrmica* ant nests).

Variable	β	s.e.m	BCa CI	
			25%	75%
Active bird nests during incubation vs. ant nests (n = 18 each)				
Intercept	3.29	1.71	− 0.02	6.82
Mean daily ambient temperature (°C)	**0.72**	**0.09**	**0.53**	**0.89**
Nest type (active bird nest)	**1.37**	**0.47**	**0.48**	**2.36**
Bird nests containing nestlings vs. ant nests (n = 9 each)				
Intercept	− 2.08	7.28	− 17.82	11.57
Mean daily ambient temperature (°C)	**0.98**	**0.37**	**0.29**	**1.78**
Nest type (active bird nest)	**6.22**	**1.04**	**4.02**	**8.20**

The presented regression coefficients were used to calculate the predicted temperatures shown in Fig. 2. The statistically significant results are highlighted with bold font. Bias-corrected and accelerated bootstrap confidence intervals (BCa CI) are shown.

During the period when most wood warblers in the population were incubating their eggs (13–25 May 2019), the mean daily ambient temperatures ranged between 9 and 20 °C (mean 15.6 °C). During this period, the predicted temperatures for active nests of birds exceeded the threshold for larval development of 16 °C for 54% of the 13-day wood warbler egg-incubation period (Fig. 2), but they did not reach the optimal 20–25 °C at which *Myrmica* develop most rapidly from egg to pre-pupae^33^. In contrast, the predicted mean daily internal temperatures of ant nests exceeded the 16 °C for only 8% of the 13-day period, and never reached the optimum of 20–25 °C (Fig. 2).

In the later nestling period (1–7 June 2019), when most wood warbler nestlings were 6–12 days old, mean daily ambient temperatures varied between 17 °C and 21 °C (mean 19.5 °C). In these conditions, the predicted temperatures of active bird nests containing older nestlings were always within the optimal range of 20–25 °C (Fig. 2), whereas the predicted mean daily temperatures in the ant nests never reached this optimum, although they often exceeded the minimum threshold of 16 °C.

**Figure 3:**
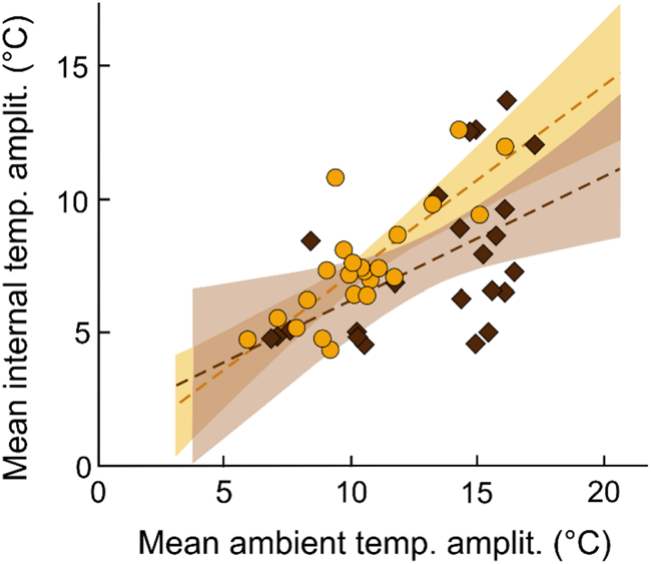
The changes of mean daily internal temperature amplitude (the difference between maximum and minimum mean hourly temperatures in a day) in relationship to mean daily ambient temperature amplitude in vacant nests of wood warblers (yellow circle, n = 23) and in occupied *Myrmica* ant nests (brown diamond, n = 23) in 2019. Dashed lines show trends and shaded areas are 95% confidence intervals.

Nevertheless, the structure of the wood warbler nests insulated less efficiently than *Myrmica* ant nests. The amplitude between daily temperature minima and maxima tended to be greater within the vacant bird nests than in ant nests (Fig. 3; Table 3), although the internal daily temperature amplitudes strongly increased with increasing ambient amplitudes in all cases (Fig. 3; Table 3).

**Table 3:** Results of bootstrapped (2000 replications) linear regressions showing the changes of mean daily internal temperature amplitude (response variable) with main effects of the respective ambient amplitude and nest type (vacant nests of wood warblers vs. *Myrmica* ant nests; n = 23 each).

Variable	β	s.e.m	BCa CI	
			25%	75%
Intercept	0.37	1.21	− 1.92	2.91
Mean daily ambient temperature amplitude (°C)	**0.55**	**0.10**	**0.36**	**0.74**
Nest type (vacant bird nest)	**1.32**	**0.58**	**0.21**	**2.42**

The statistically significant results are highlighted with bold font. Bias-corrected and accelerated bootstrap confidence intervals (BCa CI) are shown.

### Ant attraction to experimentally heated and unheated bird nests

To test experimentally whether the heat or the nest structure itself attracted ants to colonise bird nests, we placed 80 artificial bird nests c. 30 cm from *Myrmica* ant nests. Among the 80 ant nests that were used in the experiments, 41 belonged to *Myrmica rubra*, 38 to *M. ruginodis* and one to *M. sabuleti*. The artificial nests resembled wood warbler nests (domed structure made of grass, leaves and moss) and contained active and inactive heating pads. Active heating pads increased the mean daily temperature of nest walls by a mean of 4.5 °C relative to outside, mimicking the thermal conditions that ants could experience in active wood warbler nests containing older nestlings (see above).

We found that ants colonised the artificial bird nests containing active heating pads much more frequently than the control nests with inactive heating pads; ant broods occurred in 68% of 31 heated and in 6% of 49 unheated nests (χ^2^ = 34.3, df = 1, *P* < 0.001). All artificial bird nests that contained the broods involved the ant species whose colony was situated closest to the artificial nest placement, comprising *M. ruginodis* (12 nests) and *M. rubra* (11), except for one nest with *L. platythorax* brood. In eight cases, ant broods disappeared from the original location after appearing in the artificial bird nests, suggesting complete translocation. Otherwise, some larvae or pupae were present both in artificial nests and the original locations, indicating partial translocation.

## Discussion

Our work is the most comprehensive study to date that examines the role of nesting birds as thermal ecosystem engineers for nest-dwelling arthropods. We demonstrate for the first time that the heat actively generated by breeding birds within their nests attracts ants to colonise these structures, likely because the insects can raise their larvae there under more suitable thermal conditions than in their own nests.

The nests built and occupied by wood warblers formed ‘heat islands’, which appeared to be warmest during the late nestling period or on cold days. As expected, the warming of the nest walls was a by-product of the endothermic activity of the birds, which generated body heat in a homeostatic manner to maintain themselves or the temperature of their eggs for normal embryo development^15–17,31,34^. A similar situation to that which we observed in wood warbler nests could also exist in many other songbirds. This is because the mean temperature of egg incubation is similar for most bird species, ranging between 30 and 40 °C^35^, with a resting body temperature of 36.0 to 40.8 °C^36^. Therefore, active nests of songbirds could be a common and globally widespread resource of warm habitats, available to ants and other nest-dwelling invertebrates. As reproduction and activity of invertebrates is temperature dependent (e.g.^37,38^), the opportunity to use warm nests of birds may promote interspecific interactions in cool areas, where the availability of warm sites is limited.

Similar to the study system of Sinclair and Chown^19^, which involved Marion flightless moths and wandering albatrosses, we found that active nests of wood warblers provided ants with advantageous thermal conditions for the survival and development of their larvae. In our study, the predicted mean daily temperatures of active nests of birds often exceeded the minimum temperature threshold of 16 °C that enables larval development of *M. ruginodis* or *M. rubra*, and is broadly similar to that of other ant species^39–42^. When large nestlings were present, the active nests of birds were the only places examined where the 20–25 °C optimum range for ant larval development could be found. In contrast, the predicted temperatures in *Myrmica* ants’ own nests never reached the optimum temperatures of 20–25 °C and exceeded the minimum 16 °C threshold only during warm weather, usually in late spring. These findings show that the survival and larval development of *Myrmica* ants was likely limited by cool weather and unfavourable thermal conditions in the ant nests, but it could be enhanced within the much warmer bird nests that were actively heated by their owners. However, contrary to our expectations, empty wood warbler nests provided poorer insulation against ambient temperatures than nearby ant nests, so raising ant broods in stable thermal conditions was more achievable in the latter. Therefore, the presence of heat and not the physical properties of bird nests alone appeared to be the imperative for the colonisation of bird nests by *Myrmica* ants, as we confirmed experimentally.

While we did not measure the development rates or survival of ant broods, the temperature ranges that we demonstrated in bird nests have been shown to enhance larval development. Consequently, the advantageous temperatures in active bird nests may have a positive effect on colony fitness through increased production of workers and queens^33,39,43^. Moreover, selection for a behaviour of moving broods between sites must involve benefits that outweigh potential costs of brood translocation^42,44,45^. Thus, frequent colonisation of heated bird nests may suggest an advantage for ants in relocating their broods there. Unlike some other social insects, many ant species are unable to regulate their nest temperature by actively generating metabolic heat or by clustering. Instead, they rely on the thermal conditions of their nests and are dependent on solar radiation and warm ambient temperatures^30^. Thus, access to warm nest locations, such as active bird nests, would help ant colonies to maintain high productivity in cool weather.

Broods of various ant species have been reported in the nests of different open- and cavity-nesting bird species^21–28^, mostly in cool regions of Europe and the Americas. Although the process of nest colonisation by ants has previously been unexamined, the mechanism that we demonstrate of ants attracted to the warmth of bird nests could be widespread in cool, temperate regions of the world, or at high altitudes in the tropics.

The benefits of colonising warm nests of birds could also be exploited by other arthropods, such as the various moths, mites, flies or fleas, the immature stages of which are often found within bird nest structures (e.g.^7,8,12,46,47^). For example, although moth larvae are often associated with concentrated food availability within bird nests, such as organic remains containing keratin (^46^ and references therein), access to a warm microclimate in nests heated by occupying birds could be another, overlooked advantage. Similar relationships may exist among the communities of specialised arthropods inhabiting the nests of mammals as well, particularly those that construct bird-like nests in burrows and arboreal situations^48,49^. These endothermic animals may also act as thermal engineers for arthropods in the insulated nests of organic materials in cool environments, in a similar manner to the wood warblers in our study.

The thermal ecosystem engineering by endothermic animals, such as songbirds, may promote commensal or mutualistic relationships with ants or other arthropods, where one or both sides of the system benefit from the cohabitation. Whilst arthropods could gain a selective advantage from the heat available in the bird nests in our study, the costs and benefits to wood warblers or other songbirds in sharing their nest with invertebrates are less clear. Ants, like other predators, such as spiders or mites, might provide nest sanitation for birds by reducing numbers of ectoparasites^22,24,29,50,51^. Although ants may also occasionally kill nestlings^29,52,53^, no cases of ant aggression toward the hosts were observed during this study. Even when ants were disturbed by observers, and defensive workers emerged from the warbler nests, the nestlings inside remained unharmed and adult birds showed no sign of distress. This lack of aggression by ants towards their hosts, and evident tolerance of the ants by the birds, was also found by Lambrechts et al*.*^24^ and supports a commensal or mutualistic relationship between these taxa.

The relationship between birds and ants, or other nest-dwelling arthropods, are likely to be facultative however, as the exploitation of warm microclimates in bird nests is only possible for the short duration of the birds’ occupation while breeding. Additionally, only some arthropods may have access to nearby bird nests, which may be used temporarily when the thermal conditions are preferable to other locations. For example, the colonisation of bird nests by ants should be most beneficial mainly in cool weather and/or during the nestling period. This is when the nests are warmed most intensively by the birds (see also^16,17,31,32,54^), representing the warmest potential nest locations for ants. Likewise, ants would be expected to abandon bird nests when the thermal conditions deteriorate, as the heat source disappears when nestlings leave or are depredated^42,44^.

Although the mobile behaviour of ants could facilitate their colonisation of bird nests^22^, it could also explain the infrequent records in the literature of ant broods in bird nests^21–28^. Ant larvae or pupae can only be detected in the nest material by thorough examination, which is generally not possible until after the birds, and the heat source, have disappeared. As such, ant broods are likely to have been under-recorded in bird nests.

In conclusion, our research is novel in explaining the mechanism underpinning the colonisation of bird nests by ants, with the presence of heat generated by the hosts playing a pivotal role. Other potential advantages for ants of using bird nests are also possible^24,26^, as are potential benefits for the birds^22,29,50^. Thus, further research is vital to fully understand the factors and mechanisms unpinning the interactions between nesting birds and reproducing ants. The thermal ecosystem engineering by birds that we present in this study might be important for the reproduction of various other arthropods inhabiting bird nests. Similar interspecific relationships could be globally widespread among birds and mammals, but may have been overlooked. As such, we encourage researchers in different climatic zones and studying different arthropod and vertebrate taxa to explore this topic and help define the geographical range and nature of the relationships between endothermic animals and nest-dwelling arthropods.

## Material and methods

### Study site

We conducted the study in the Białowieża Forest, eastern Poland (coordinates of Białowieża village: 52°42′N, 23°52′E), a c. 1500 km^2^ woodland area straddling the Polish-Belarusian border. The climate is subcontinental with mean temperatures during May–July of 13–18 °C^55,56^.

We carried out observations mainly in Białowieża National Park (hereafter BNP) within permanent study plots totalling c. 100 ha^57,58^ or in other fragments of primeval oak-lime-hornbeam stands, which is the main habitat of wood warblers in BNP^59^. The experiments were performed in managed oak-lime-hornbeam forest adjoining BNP.

### Nest searches and inspections

The wood warbler is a small migratory songbird (c. 10 g) that builds dome-shaped nests (c. 15 cm diameter) of woven grass, leaves and moss, lined with animal hair, and placed among leaf litter or sparse vegetation on the floor of temperate Eurasian forests^20,60^. Egg-laying occurs from late April until early July, with first clutches usually containing 6–7 eggs incubated for 12–13 days until hatching. The nestling period lasts a further 12–13 days until fledging, when the nest is vacated^20,59^.

We searched for wood warbler nests in May–June 2017–2019 by following birds, mainly during nest-building, and monitoring the nests every 1–6 days to establish the dates of egg laying, hatching (day 0), nestlings leaving the nest, or nest failure (death of embryos or nestlings^59^).

*Myrmica* ant nests were located in 2019, within c. 20–30 m from sampled wood warbler nests, by carefully inspecting potential sites on the forest floor among fallen deadwood, patches of moss or tussocks of grass^61^. Most *Myrmica* ants form colonies, i.e. groups of ants living together in the same nest structure, containing 200–500 workers, although colonies of *Myrmica rubra* often contain 1000 workers^62^. When locating ant nests, we gave special attention to leaving the nest structure intact to prevent brood relocation by ant workers, and to allow unbiased temperature measurements. In most cases, ant broods or workers were present in the same location in the following days, indicating minimal disturbance^45^.

### Temperature measurements

We measured the temperature of wood warbler nests in 2017–2019. To check if nest temperature was elevated due to heating by the birds, we took measurements from active nests and repeated them in the same nests after they had been vacated by nestlings or depredated, if the nest structure remained intact. To check if the heating effect increased over the nesting cycle of birds, we took measurements twice from active nests: during the egg incubation when a single adult warms the nest contents, and in the nestling period when typically six feathered nestlings were 6–11 (median 7) days old^54,59^. We also took temperature measurements from within ant nests in 2019 to compare their thermal conditions with the active and vacant bird nests.

We were unable to take the measurements at the same ambient temperatures in all bird and ant nests, due to the limited number of the dataloggers available and high losses of bird nests due to predation. Therefore, to account for variation in ambient conditions between sampling days, we measured temperatures simultaneously inside and outside of each bird or ant nest. Nest temperatures were recorded automatically using Voltcraft DL-111 K temperature loggers (accuracy ± 1 °C)^63^. Ambient temperatures were recorded using iButtons DS1922L (accuracy ± 0.5 °C in the operating range from − 10 °C to + 65 °C)^64^. Both types of loggers were calibrated prior to measurements, showing an acceptable mean temperature difference of 0.26 °C.

We programmed each set of one internal and one external logger to record temperature values simultaneously every 5 min from the approximate time of installation at a nest. We mounted internal and external loggers together at each nest. During the measurements, a sensor (c. 1 mm diameter) was inserted into the side walls of the bird nest, where ants placed their broods^21^, or among ant larvae or pupae inside *Myrmica* ant nests. We mounted the external logger approximately 10 cm above the ground, several metres from the bird or ant nest. The external logger was placed within a tubular white plastic sleeve of c. 7 cm diameter that was open at both ends to allow air movement while shading the sensor from direct sunlight. The mean daily temperatures recorded by external loggers closely corresponded to values recorded by the local meteorological station at BNP (r_S_ = 0.91, *P* < 0.001). We retrieved pairs of data loggers together from the field for data extraction after a minimum 25 h of temperature recording.

### Experiments with artificial nests of birds

To test whether heat or the nest structure itself attracts ants to colonise bird nests, we performed field experiments with heated and control (unheated) artificial nests that mimicked the natural nests of wood warblers. The dome-shaped artificial nests were made of grass (67%), leaves (23%) and moss (10%) in proportions and mass (20 g in total) approximating a wood warbler nest in BNP (Maziarz M. and Hebda G., unpubl. data). We loosely bound the nests with cotton thread to maintain their structural integrity during transportation.

We conducted the experiments on 4–20 July 2019 in two areas c. 500 m apart. Prior to experiments, we searched for *Myrmica* ant nests in sampling locations distributed 20–40 m apart and marked ant nests with tags. We lightly sprayed artificial nests with water just before their installation in the field to provide ants with similar humid conditions to natural sites. Each artificial nest was then placed c. 30 cm from the ant nest, allowing easy brood relocation if the ants chose to do so. We placed each artificial nest in a shallow depression in the soil made by the observer, and camouflaged it with leaves to mimic a natural wood warbler nest^60^. We inserted heating pads (Aqua Pack ‘heat pack 40 h’) into all artificial nests. Half of the nests contained an activated pad that generated heat of approximately 40 °C through oxidation of iron powder, while the remaining half served as a control with inactive pads. We repeated the experimental procedure four times with 20 ant nests tested per day, sampling a total of 80 ant colonies comprising originally 40 with heated artificial nests and 40 controls. Each ant colony was sampled once, at a mean daily ambient temperature of around 14 °C or 18 °C. Despite different ambient temperatures, the nest colonisation rate by ants remained similar between sampling days (Supplementary Table S1). To determine whether ants colonised artificial bird nests by relocating their broods to them, we inspected the nests the following day, at least 24 h from installation.

To confirm that active heating pads replicated the body heat of birds warming up the nests from within, we measured the temperature of the walls of artificially heated nests, following the protocol used at natural nests of birds (see above). As the mean daily internal-ambient temperature difference was less than 0.5 °C in nine of the nests, indicating failure of their heating pads, we pooled these nine nests with the controls, giving a final 49 unheated nests and 31 heated nests for analysis.

### Ant specimen identification

For specialist entomological identification, we collected five to ten ant specimens from the original ant nests used for the temperature measurements, after their completion. Similarly, we collected ant specimens from the ant nests tested during the experiments with artificial nests, as well as those that colonised the artificial nests.

### Data analyses

In the analyses, we used samples of nests for which full pairs of temperature measurements were available, comprising: the same bird nests when containing incubated eggs and when vacant (n = 44), or the same nests containing nestlings and when vacant (n = 29), bird nests containing incubated eggs and nearby ant nests (n = 18), bird nests with nestlings and nearby ant nests (n = 9), or vacant bird nests and nearby ant nests (n = 23 each), the latter belonging to *Myrmica ruginodis* (13 nests) or *M. rubra* (ten).

To assess the thermal conditions of natural nests of birds and ant nests, from each temperature recording we selected a 24-h sequence and calculated hourly means to define the mean, minimum and maximum daily temperature, and also the daily amplitude, i.e. the difference between maximum and minimum hourly mean temperature in a day.

For comparisons between temperatures of bird nests when they were active and vacant, we standardised observed internal temperature values to varying ambient conditions by using ‘temperature differences’, derived by subtracting mean daily ambient values from the corresponding nest readings^54^. We evaluated how much the heating activity of birds increased the nest temperatures by comparing the internal-ambient temperature differences between active and vacant nests, using paired t-tests.

To test if the heating activity of birds was related to ambient temperatures, we used linear regression models containing mean daily internal temperature as the response variable, with covariates of mean daily ambient temperature and nest type (active vs. vacant nests) as main effects, including an interaction term when necessary. However, as the temperature was recorded repeatedly in the same bird nests at different nesting stages, we first tested for non-independence of the measurements. For this purpose, we performed mixed-effect linear models with a random intercept of the nest identity and compared the fit of these models with the corresponding linear regressions, using generalized least squares fit by maximum likelihood in the nlme package for R^65^. As the likelihood ratio (L) was < 1.5 and *P* value > 0.2, indicating a non-significant random effect, we presented only the results of the linear regression models. Similarly, using the generalized least squares, we compared the models’ fit by maximum likelihood with and without the interaction terms, which were retained only if this significantly improved the model fit.

To check if active bird nests provided more advantageous temperatures than the ants’ own nests, we estimated bird and ant nest temperatures for the same ambient conditions. Weather data obtained from the meteorological station in Białowieża village provided a continuous daily record of temperature values. We used the mean daily ambient temperatures from two nesting periods corresponding to median dates of the 13-day period of egg incubation and when nestlings were 6–12 days old in the majority of 108 wood warbler nests monitored in BNP during 2019 (Maziarz M.*,* unpubl. data). The predictions were based on the coefficients obtained from linear regression models, after assuring for non-independence of the measurements at active nests of birds and ant nests found nearby, following the protocol described above. The models included mean daily nest temperature as a response variable and the main (fixed) effects of mean daily ambient temperature and nest type (bird vs. ant nest) as covariates. We performed modelling separately for bird nests containing incubated eggs or nestlings. Based on the predicted values for the two bird nesting periods, we calculated the number of days when ant broods present in ant nests or in active bird nests would experience conditions above the lower developmental threshold of 16 °C, i.e. the minimum temperature for development of *M. ruginodis* or *M. rubra* larvae. We also calculated the number of days when the optimal temperatures of 20–25 °C occurred, at which these *Myrmica* develop most rapidly from egg to pre-pupae^33^.

Additionally, we used linear regression models to test if vacant bird nests insulated more effectively than ant nests. To account for the daily variation in ambient temperatures which may affect nest temperatures, the models contained mean daily internal temperature amplitude as a response variable, and the covariates of the respective ambient amplitude and nest type (ant nest vs vacant bird nest) as main effects.

In all cases we calculated coefficients and 95% confidence intervals using bootstrapping (2000 replications; boot package for R^66,67^).

To test if ants more frequently colonised heated than control artificial bird nests, we compared the proportions of colonised and uncolonised nests using two-tailed χ^2^ tests.

All statistical calculations followed formulae in R version 3.4.4^68^.

## Supplementary information

Supplementary information.

## Data Availability

The datasets generated during and/or analysed during the current study are available from the corresponding author on reasonable request.
