# Supplementary material for: Thermal ecosystem engineering by songbirds promotes a symbiotic relationship with ants
Source: Sci Rep. 2020 Nov 23;10:20330. doi: 10.1038/s41598-020-77360-z (PMC7684287; doi:10.1038/s41598-020-77360-z)
Supplement: Supplementary file 1 — Supplementary information. [file 41598_2020_77360_MOESM1_ESM.pdf]

Title: Thermal ecosystem engineering by songbirds promotes a symbiotic relationship with ants

Authors: Marta Maziarz [1], Richard K. Broughton [2], Luca Pietro Casacci [3], Anna Dubiec [1], István Maák [4], Magdalena Witek [1]

[1] *Museum and Institute of Zoology, Polish Academy of Sciences, Wilcza 64, 00-679 Warsaw, Poland*

[2] *UK Centre for Ecology & Hydrology, Maclean Building, Benson Lane, Crowmarsh Gifford, Wallingford, OX10 8BB, UK*

[3] *Department of Life Sciences and Systems Biology, University of Turin, Via Accademia Albertina 13, 10123 Turin, Italy*

[4] *Department of Ecology, University of Szeged, Közép fasor 52, Szeged 6726, Hungary*

Supplementary Table S1 The number of artificial bird nests that were heated or unheated (control) and colonised or not colonised by *Myrmica* ants to raise their broods. Heated nests contained activated heating pads to mimic the body heat of birds, and control nests contained inactive pads (see Methods). The table shows the results of experiments performed on 4-20 July 2019 in two areas c. 500 m apart in managed oak-lime-hornbeam forest adjoining Białowieża National Park, Poland. The ambient temperatures shown are the mean daily values recorded during the experiments, obtained from the meteorological station in the Białowieża village. In nine nests originally containing active heating pads, the difference between the mean daily internal and ambient temperature was less than 0.5 °C, indicating failure of these nine pads. Thus, these nests have been pooled with the controls to show the occupation rate of effectively heated artificial nests in the Results.

Area	Date	Ambient temperature (°C)	Nest type	N nests with ant broods	N nests without ant broods
A	4-5 July	13.7-14.5	heated	6	2
			unheated	0	12
B	5-6 July	13.7-14.5	heated	4	3
			unheated	1	12
A	6-7 July	14.5-16.5	heated	6	2
			unheated	2	10
B	19-20 July	17.8-18.5	heated	5	3
			unheated	0	12
